# Prolonged intermittent theta burst stimulation for post-stroke aphasia: protocol of a randomized, double-blinded, sham-controlled trial

**DOI:** 10.3389/fneur.2024.1348862

**Published:** 2024-04-25

**Authors:** Ying Liu, Jingdu Zhao, Zhiqing Tang, Yikuang Hsien, Kaiyue Han, Lei Shan, Xiaonian Zhang, Hao Zhang

**Affiliations:** ^1^School of Rehabilitation, Capital Medical University, Beijing, China; ^2^Beijing Bo'ai Hospital, China Rehabilitation Research Center, Beijing, China; ^3^Cheeloo College of Medicine, Shandong University, Jinan, Shandong, China; ^4^School of Life and Health Sciences, University of Health and Rehabilitation Sciences, Qingdao, China

**Keywords:** study protocol, post-stroke aphasia, supplementary motor area, prolonged intermittent theta burst stimulation, event-related potentials, brain-derived neurotrophic factor

## Abstract

**Background:**

Post-stroke aphasia (PSA) is one of the most devastating symptoms after stroke, yet limited treatment options are available. Prolonged intermittent theta burst stimulation (piTBS) is a promising therapy for PSA. However, its efficacy remains unclear. Therefore, we aim to investigate the efficacy of piTBS over the left supplementary motor area (SMA) in improving language function for PSA patients and further explore the mechanism of language recovery.

**Methods:**

This is a randomized, double-blinded, sham-controlled trial. A total of 30 PSA patients will be randomly allocated to receive either piTBS stimulation or sham stimulation for 15 sessions over a period of 3 weeks. The primary outcome is the Western Aphasia Battery Revised (WAB-R) changes after treatment. The secondary outcomes include The Stroke and Aphasia Quality of Life Scale (SAQOL-39 g), resting-state electroencephalogram (resting-state EEG), Event-related potentials (ERP), brain derived neurotrophic factor (BDNF). These outcome measures are assessed before treatment, after treatment, and at 4-weeks follow up. This study was registered in Chinese Clinical Trial Registry (No. ChiCTR23000203238).

**Discussion:**

This study protocol is promising for improving language in PSA patients. Resting-state EEG, ERP, and blood examination can be used to explore the neural mechanisms of PSA treatment with piTBS.

**Clinical trial registration:**

https://www.chictr.org.cn/index.html, ChiCTR2300074533.

## 1 Introduction

Stroke is the second leading cause of death and a major cause of disability worldwide (1). Aphasia is a functional impairment after a stroke that affects almost one-third of stroke survivors for acute and rehabilitation settings (2). Post-stroke aphasia (PSA) has a significant effect on patients including social interactions, depression, and lower quality of life (3–6). PSA treatment strategies include behavioral intervention, pharmaceutical interventions, noninvasive brain stimulation (7). Conventional speech and language therapy (SLT) is the mainstay of treatment, but the efficacy of SLT is not as good as it should be (8–10). And there is no consistent evidence that any medication has substantial effect on aphasia recovery (11). Non-invasive brain stimulation therapy has attracted increasing attention as a complementary treatment for PSA (12).

By generating induced electrical currents through the magnetic field that passes through the skull, transcranial magnetic stimulation (TMS) affects the excitability of the cerebral cortex (13). Intermittent theta burst stimulation (iTBS), a specific modality of TMS, offers the advantage of rapid administration and can be well-tolerated, making it a feasible intervention for stroke recovery (14–16). And it has shown to be promising in modulating cortical excitability and inducing neuroplastic changes in specific brain regions involved in language processing (17). Previous studies have consistently shown that iTBS is a valuable treatment modality for enhancing language function and inducing cortical plasticity in human brain networks over the short- and intermediate-term (15, 18). However, it is important to note that the degree of improvement in language scores has been relatively modest, and certain clinical trials have reported improvement limited to specific aspects, such as comprehension (19). Therefore, there is an urgent need to improve the overall therapeutic efficacy of iTBS in PSA. Possible reasons affecting treatment efficacy include dose and target of stimulation.

Most iTBS studies have used 600 pulses (18, 20), but study showed that increasing iTBS stimulation dose resulted in dose-dependent effects at the local level (cortical excitability) (21). The effect of three consecutive 600 pulses iTBS, named prolonged iTBS (piTBS), on cortical excitability was the most significant (21). Therefore, piTBS has also attracted much attention in recent years. For example, a study comparing different doses and modes of theta burst stimulation for refractory depression founded that piTBS were effective in improving depressive symptoms (22). Significant improvements were also seen in veterans with mild alcohol use disorder (23).

Another important factor affecting efficacy is the stimulation target. Classical language centers include Broca’s and Wernicke’s areas and their homologs in the right hemisphere continue to dominate the selection of target locations for TMS treatment of aphasia (18, 19, 24). Therapeutic efficacy based on these target targets remains limited. The supplementary motor area (SMA) has been neglected in speech and language processing, but recent research has shown that it has a superior control function during speech communication and language reception (25, 26). And it is particularly important when task demands increase, including a functional division of labor between different subregions in speech motor and cognitive processing (27). It has also been shown that PSA patients have reduced spontaneous brain activity in the left SMA (28). Transcranial alternating current stimulation on SMA targets significantly improves language comprehension (29). To date, there are no studies related to piTBS treatment based on SMA target.

Furthermore, the mechanism of piTBS to promote language recovery is not clear. Electroencephalogram (EEG) can be used to obtain temporal information on speech production, thus revealing the internal processing of language and compensating for the limitations of behavioral testing (30). The N400 is an electrophysiological indicator related to semantic processing. Compared to healthy controls and individuals with other types of brain damage, PSA patients typically exhibit diminished or absent amplitudes, prolonged latency, and irregular scalp distribution (31, 32). In a picture-text matching task, they reported an abnormal shift of the centro-parietal N400 waveform from the right hemisphere to the more lateral left hemisphere after treatment (33). The mismatch negativity (MMN), as an electrophysiologic index related to attentional and perceptual processing, has also been widely used in the assessment of PSA. Researchers founded an increase in MMN amplitude after treatment, which could confirm changes in neuroplasticity at the structural and lexical level, correlating with improvements in clinical symptoms in PSA patients (34). Abnormalities in the above two components are suggestive of their difficulties in processing and comprehending semantic information. Further studies will help to explore the electrophysiologic characteristics in PSA.

As a neuronal modulator, brain-derived nerve growth factor (BDNF) has been shown to be an important upstream regulator of hippocampal and neocortical long-term potentiation (LTP) during motor learning (35). BDNF can also be modulated by therapeutic interventions, with BDNF levels almost tripling in subjects receiving TMS compared to healthy subjects receiving sham stimulation (36). In addition, BDNF is increased in depressed patients who receive multiple TMS treatments (37). Therefore, we hypothesized that similar manifestations would be seen in PSA and that piTBS might alter the level of BDNF, which plays an important role in aphasia recovery.

In conclusion, this study aims to investigate the efficacy of piTBS targeting the left SMA in PSA patients, and to explore the underlying mechanisms facilitating language recovery. Through this study, we hope to provide new treatment strategies for clinical practice.

## 2 Methods

### 2.1 Study design

**Figure 1:**
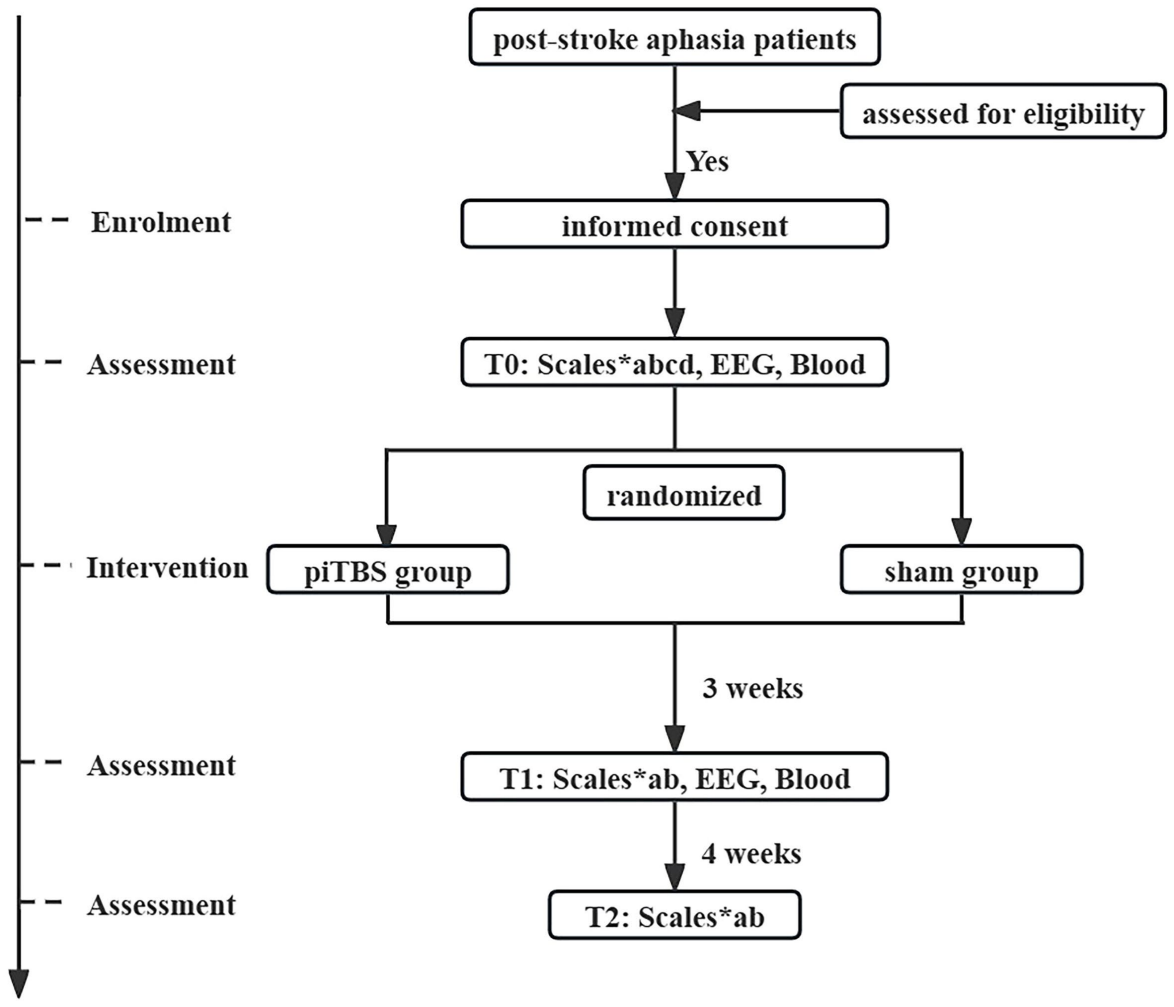
Flow chart of study procedure. EEG, electroencephalogram; T0, before treatment; T1, after treatment; T2, at 4-weeks follow up; a, Western Aphasia Battery Revised; b, The Stroke and Aphasia Quality of Life Scale; c, The Edinburgh Handedness Inventory; d, The National Institutes of Health Stroke Scale.

This is a randomized, double-blinded, sham-controlled trial. The study will adhere to the ethical principles outlined in the Declaration of Helsinki and will follow the Consolidated Standards of Reporting Trials (CONSORT) guidelines (38), as well as the Recommendations for Interventional Trials (SPIRIT) (39). All patients who meet the inclusion and exclusion criteria will be randomly assigned in a 1:1 ratio to the following piTBS group and sham group after obtaining written informed consent. All patients will undergo corresponding assessments before treatment (T0), after treatment (T1), as well as during the 4-weeks follow up (T2). Please refer to Figure 1 and Table 1 for the specific study process. This study has been approved by the Medical Ethics Committee of the China Rehabilitation Research Center (No.2023-027-2) and registered in the Chinese Clinical Trial Registry (No. ChiCTR2300074533).

**Table 1:** Study process schedule.

	Pre-enrollment	T0	Intervention	T1	T2
	−7 days	Day 0		±3 days	±3 days
Screening	√				
Informed consent	√				
Random allocation		√			
Demographic information		√			
EHI		√			
NIHSS		√			
SAQOL-39 g		√		√	√
WAB-R		√		√	√
Resting-state EEG		√		√	
ERP		√		√	
Blood		√		√	
Adverse event			√	√	

T0, before treatment; T1, after treatment; T2, at 4-weeks follow up.

### 2.2 Participants

#### 2.2.1 Inclusion criteria

- The patients were diagnosed with ischemic or hemorrhagic stroke with the lesion located in the left hemisphere, with a disease duration ranging from 1 to 12 months;
- The patients will undergo assessment for aphasia using the Western Aphasia Battery Revised (WAB-R), and the WAB-R aphasia quotient will be less than 93.8 points;
- Patients aged 18–75 years;
- Right hand;
- Normal language function before the onset of the disease;
- The native language is Chinese, primary school education or above (with more than 6 years of education).

#### 2.2.2 Exclusion criteria

- Aphasia caused by brain tumor, traumatic brain injury or other diseases;
- Contraindications to TMS treatment such as pacemakers, cochlear implants, or other metallic foreign bodies and any electronic devices implanted in the body;
- Combined history of epilepsy;
- Patients with severe cardiac, pulmonary, hepatic, renal, and other systemic diseases that cannot be controlled by conventional medication, as detected by laboratory examinations;
- Presence of a history of comorbid alcohol, drug, and other abuse;
- Patients who do not meet the inclusion criteria due to the presence of other examination abnormalities;
- Women of childbearing age who are pregnant or planning to become pregnant;
- Patients who have previously undergone TMS or transcranial electrical stimulation (TES) within 3 months before enrollment;
- Patients who are participating in other clinical research trials.

#### 2.2.3 Withdrawal criteria

- Severe adverse reactions or complications, such as persistent headaches, seizures, or other discomfort related to TMS;
- Protocol violations: Patients unable to comply with the study protocol, such as refuse to undergo assessments or interruption of treatment for more than 3 consecutive days;
- Development of new health issues or worsening of existing conditions deemed inappropriate for continued participation in the trial;
- Use of other TMS or TES treatments;
- Withdrawal from the trial without any specific reason.

#### 2.2.4 Sample size

Based on the preliminary experimental results, the effect size [f(V)] for the piTBS group compared to the sham group in the primary outcomes is 0.25. With a significance level (α) of 0.05 and a power (1-β) of 0.95, considering a two-group design involving before and after treatment, and during follow-up assessments conducted at three time points, the anticipated sample size calculated using G*Power 3.1.9.7 for the repeated measures ANOVA model was 28. Anticipating a dropout/exclusion rate of around 10%, a total of 30 patients will need to be enrolled, with 15 patients allocated to each group.

### 2.3 Randomization and blinding

The randomized sequence of numbers generated by SPSS software will be written in order and placed in opaque sealed envelopes. All eligible patients will be randomly assigned to one of the two treatment groups in the order of enrollment. Recruiters, study physicians, TMS operators, clinical staff, evaluators, data collectors and statistical analysts are blinded. These people will not know the group of patients.

### 2.4 Interventions

The patients will be assigned under the 1:1 randomization formula, constituting two groups for the present study: 15 patients treated with piTBS +15 patients treated with sham stimulation. Furthermore, a designated speech rehabilitation therapist will assess the scale evaluations of all enrolled patients. A transcranial magnetic stimulator (Neurosoft, YD-MT500, Russia) with a figure-of-eight coil and a sham stimulation coil will be used. The coil position targeting the SMA is determined using the international 10–20 EEG system as a reference. The target stimulation site is set at 15% of the nasion-inion distance anterior to the Cz position, along the sagittal midline (40). The coil is positioned to form a 45°with the sagittal plane, in order to deliver stimulation to the left SMA.

#### 2.4.1 Resting motor threshold

Patients will be instructed to attain a state of relaxation while maintaining their eyes open (41). The RMT is defined as the ability to induce Motor Evoked Potentials (MEPs) of no less than 50 millivolts in at least five out of 10 consecutive stimulations, measured at the right primary motor cortex.

#### 2.4.2 Protocol

- piTBS group: the piTBS will be adopted based on the standard iTBS protocol (a 2 s train of TBS repeated every 10 s at an intensity of 80% RMT and a total of 600 pulses) by increasing the number of pulses to 1,800. This will spend 9 min 42 s every session.
- Sham group: the sham group will receive treatment with the same target coordinates, parameters, dosage, but as sham coils.

#### 2.4.3 Routine medical care

All patients will receive standard medication and rehabilitation therapy. Additionally, they will undergo speech and language therapy (SLT) for 1 h daily following treatment, five times a week, for 3 weeks. The training program is based on the Schuell’s stimulation approach, focusing primarily on language comprehension and expression.

### 2.5 Outcomes

#### 2.5.1 Behavioral assessments

##### 2.5.1.1 Western Aphasia Battery Revised

The Western aphasia battery revised (WAB-R) is a standardized tool used to assess post-stroke aphasia. The aphasia quotient (AQ) is derived by adding the standardized scores from the WAB-R subtests of spontaneous speech, auditory comprehension, repetition, and naming. The AQ provides a composite index of language and communication skills, which less than 93.8 indicate aphasia (42).

##### 2.5.1.2 The Stroke and Aphasia Quality of Life Scale

The Stroke and Aphasia Quality of Life Scale (SAQOL-39 g) is a self-report questionnaire specifically developed to evaluate the health-related quality of life in PSA patients (43, 44). The SAQOL-39 g consists of 3 dimensions: physical, communicative, and psychosocial. The physical dimension consists of 16 items, the communicative dimension consists of 7 items, and the psychosocial dimension consists of 16 items, for a total of 39 items. All items are rated on a 5-point scale, with higher scores indicating better quality of life. The SAQOL-39 g provides a brief, reliable measure of health-related quality of life that covers the sequelae of stroke.

##### 2.5.1.3 The Edinburgh Handedness Inventory

The EHI is a straightforward survey utilized for evaluating hand preference and lateralization of hand proficiency. The EHI accurately distinguishes between left, right, and mixed hand preference (45). The questionnaire comprises 10 items that inquire about the dominant hand for common unimanual activities, including writing, throwing, and using scissors. Scores are determined by operational indicators, where a score of less than −40 designates left-handedness, a score between −40 and 40 indicates mixed-handedness, and a score exceeding 40 denotes right-handedness.

##### 2.5.1.4 The National Institutes of Health Stroke Scale

The National Institutes of Health Stroke Scale (NIHSS) is a standardized tool used to quantify stroke severity (46). It evaluates impairment in 11 domains including consciousness, eye movements, visual fields, facial palsy, limb strength, sensation, coordination, language, speech, and neglect. The total NIHSS score sums all domain scores and ranges from 0 to 42, with higher totals signifying greater stroke impact.

##### 2.5.1.5 Tolerability and safety

This study will use a 16-item symptom questionnaire to rate the subjective symptom [headache, scalp pain, arm/hand pain, other pain(s), numbness/tingling, other sensation(s), weakness, loss of dexterity, vision/hearing change(s), ear ringing, nausea/vomiting, appetite loss, rash, skin change(s) or any other symptom(s)] on a scale of 0 to 5 (none, minimal, mild, moderate, marked, severe) prior to any TMS application (47). Assessments will be made at the end of each treatment session to detect the presence of any adverse events.

#### 2.5.2 Neurophysiological examinations

Patients will be seated in a comfortable chair approximately 60 cm from a computer monitor in an electrically shielded room. EEG signal will be record by the NeuroScan EEG acquisition system (64-channel EEG cap, EEG amplifiers, signal converters, and CURRY 8.0 software). Stimulation presentation will be programmed in E-Prime 3.0 (Psychology Software Tools Inc., Pittsburgh, PA). The electrodes will be positioned in accordance with the international standard EEG 10–20 system, and the resistance ≤ 10 kΩ. Patients will be instructed to remain still, silent, and refrain from extraneous movements during the recording session.

##### 2.5.2.1 Resting-state EEG

Patients will be directed to remain motionless, fixate their attention on the “+” symbol located in the screen’s center, stay alert and avoid sleepiness throughout the recording. A 10-min continuous EEG signal will be recorded.

##### 2.5.2.2 Event-related potentials

N400: The visual stimulation used for this study comprise semantically related pairs of pictures and words. The pictures are obtained from an international picture database and consisted of 120 common object images (48). Each image is sized at 300 × 300 pixels and present in Microsoft YaHei font with a font size of 24. The pictures are displayed for a duration of 1 s. Subsequently, the target word appears at the same position as the previous picture and remain on the screen for 3 s (49). Patients are instructed to judge as quickly and accurately as possible whether the target word is consistent or inconsistent with the preceding picture by pressing the left mouse button for consistent and the right mouse button for inconsistent. The longer presentation duration of the target word was to provide sufficient time for patients to make judgments and responses. Patients’ choices and reaction times were recorded by E-Prime software. MMN: The auditory stimulation is presented binaurally through insert earphones at an intensity of 70 dB sound pressure level. The stimulation consisted of 1,000 and 1,500 Hz pure tones occurring at frequencies of 90% and 10%, respectively. Each tone is presented for 50 ms. In total, 500 stimulations are delivered in each experimental block, comprising approximately 5 min and 45 s. The frequent 1,000 Hz tones is served as standard stimulation, while the infrequent 1,500 Hz tones are deviant stimulation in this auditory oddball paradigm (50).

#### 2.5.3 Neurochemical assessment

To investigate serum biomarkers related to synaptic plasticity and neuronal excitability, 5 mL blood samples will be collected from patients before and after treatment. Then those blood promptly centrifuged at 3,000 r/min for a duration of 10 min to facilitating serum separation. The resultant serum is aliquoted and preserved at −80°C for further analyses.

### 2.6 Data analysis

All data will be analyzed by professional statisticians using SPSS software (Version 25.0; IBM, Armonk, NY, United States). Baseline characteristics will be described using means ± standard deviations for continuous variables and frequencies for categorical variables. Prior to applying independent sample t-tests or Mann–Whitney U tests for continuous variables, a normality test will be conducted using either the Kolmogorov–Smirnov or Shapiro–Wilk test. Categorical variables will be compared using chi-square tests or Fisher’s exact tests. The primary outcome is the change in WAB-R scores before and after treatment. The secondary outcomes include changes in blood markers, EEG, and ERP indices before and after treatment. These measures will be analyzed using a Linear Mixed Effects Model (LMM), where group acts as a fixed effect, time points as a repeated measure, and patients as random effects within the LMM framework. If significant interaction effects between groups and time points are revealed by the LMM, post-hoc comparisons will be conducted to identify the time points at which differences occur, using methods like Bonferroni-corrected pairwise comparisons.

The acquired EEG signals will be analyzed using MATLAB2021b. The EEGLAB toolbox (version 2023.0) will be used for EEG data preprocessing (51). MMN and N400 components in the ERP data will be extracted through time-window analysis and peak detection techniques. EEG data analysis will focus on power spectral analysis to assess changes in key EEG frequency bands, such as alpha and beta bands (52). These data will be analyzed within the LMM framework to examine the patterns of change before and after treatment and their correlation with clinical improvement. If the LMM reveals significant interaction effects between groups and time points, appropriate post-hoc comparisons, such as Bonferroni-corrected pairwise comparisons, will be conducted to identify the specific time points at which differences occur.

## 3 Discussion

We aim to investigate that piTBS can effectively facilitate language recovery. Compared with repetitive TMS, we choose iTBS because of its higher spatiotemporal resolution and stronger focusing of activated neural circuits (53, 54), making it more suitable as an intervention for aphasia rehabilitation. PiTBS significantly increases the wave amplitude of MEP and promotes cortical excitability (21). In major depressive disorder, piTBS has been shown to effectively improve depressive symptoms compared to sham stimulation (22, 55). This approach has also demonstrated promising therapeutic effects in mild alcohol use disorder (23, 56). Additionally, a previous study on treating aphasia with iTBS in the ipsilateral cerebral hemisphere indicated effectiveness in enhancing language functioning (15). However, the efficacy of piTBS in treating Post-Stroke Aphasia (PSA) is not yet established. Our study is designed to address this gap by investigating the potential of piTBS in PSA patients.

The SMA, located in the medial region of Brodmann area 6, is an integral part of the expanded language network (57). The Frontal Aslant Tract (FAT), which plays a crucial role in speech and language processing, links the SMA with the Broca’s region, underscoring the anatomical and functional connectivity important for these processes (58, 59). Hemorrhage in the left supplementary motor area (SMA) may lead to various speech production and articulation issues in patients. This particularly affects the initiation of sequential articulations and the generation of spoken language output (60). Furthermore, findings from task-based functional magnetic resonance imaging studies suggest that the functionality of the SMA plays a beneficial role in language recovery after stroke (25). This is indicated by the differentiation in activation patterns related to language production and those associated with cognitive processing (25). Recent studies have demonstrated the efficacy of transcranial electrical stimulation targeting the SMA in enhancing speech comprehension among chronic PSA patients (29). However, the focus of TMS treatments has predominantly been on the inferior frontal gyrus, with limited exploration of the SMA as a potential target (61). This study aims to broaden treatment options for PSA by investigating the SMA’s role as a potential target.

TMS is a non-invasive brain stimulation technique that applies pulsed magnetic fields to the brain. This process results in either the excitation or inhibition of neurons, consequently influencing brain metabolism and neural electrical activity. EEG, another non-invasive method, is utilized to record the brain’s electrical activity. In PSA patients, monitoring power in the theta, alpha, and beta frequency bands via EEG can serve as both a repeated and a sensitive measure to assess function and detect changes in patients with chronic aphasia (62). Both N400 and MMN are components closely related to language processing, and the abnormalities in two components suggest difficulties in processing and understanding semantic information as well as reduced sensitivity and adaptability to rule violations in language (50, 63). Reduction of the N400 is associated with language improvement (31). In another longitudinal study utilizing the MMN paradigm, it’s found that the laterality index of MMN amplitudes at approximately 2 weeks post left-hemisphere stroke serve as more sensitive predictors of language outcome, among which the laterality index over the perisylvian area exhibits the best predictive value (50). However, there is a limited amount of research investigating the changes in MMN and N400 in response to TMS before and after treatment. Therefore, we anticipate that the changes in ERP will help to explain the findings and whether these results are consistent with neuroplasticity mechanisms. BDNF plays a key role in activity-dependent modifications of neuronal connectivity and synaptic strength in neuroplasticity studies (64). The magnetic-electric responses induced by TMS may affect BDNF levels in both serum and cerebrospinal fluid. It has been observed that iTBS treatment can reverse the decline in mature BDNF-related protein levels (65). Moreover, long-term iTBS has been found to promote neural structural and functional recovery by enhancing neurogenesis and migration through the miR-551b-5p/BDNF/TrkB pathway in cerebral ischemia–reperfusion injury models (66). Additionally, iTBS has been reported to induce LTP (67). In Alzheimer’s patients, improvement in patients’ cognitive function after application of TMS is associated with elevated peripheral BDNF levels (68). Low-frequency TMS therapy can improve the language function of patients with non-fluent aphasia after stroke more effectively and it also promote the expression of BDNF more effectively, thereby improving nerve repair and protecting brain tissue (69). Therefore, our study aims to explore the levels of BDNF before and after piTBS treatment. By conducting a thorough investigation into the effects of piTBS treatment on BDNF, we hope to further understand the impact of this therapy on neuroplasticity and treatment outcomes. This study can contribute to expanding our understanding of piTBS as a therapeutic method and provide more accurate guidance and improvement strategies for clinical application.

In conclusion, the primary objective of this study protocol is to investigate the effectiveness of piTBS in treating PSA patients. Furthermore, this protocol aims to explore the changes in electrophysiological characteristics and BDNF levels following piTBS intervention. These investigations are expected to provide a robust theoretical foundation for the clinical application of piTBS in the treatment of PSA.

## Data availability statement

The original contributions presented in the study are included in the article/supplementary material, further inquiries can be directed to the corresponding author.

## Ethics statement

The studies involving humans were approved by the Medical Ethics Committee of the China Rehabilitation Research Center. The studies were conducted in accordance with the local legislation and institutional requirements. Written informed consent for participation in this study was provided by the participants’ legal guardians/next of kin.

## Author contributions

YL: Conceptualization, Methodology, Writing – original draft, Writing – review & editing. JZ: Investigation, Resources, Writing – review & editing. ZT: Investigation, Resources, Writing – review & editing. YH: Software, Visualization, Writing – review & editing. KH: Data curation, Resources, Writing – review & editing. LS: Funding acquisition, Project administration, Supervision, Writing – review & editing. XZ: Project administration, Supervision, Writing – review & editing. HZ: Conceptualization, Project administration, Supervision, Writing – review & editing.

## References

[1] Katan M Luft A. Global burden of stroke. Semin Neurol. (2018) 38:208–11. doi: 10.1055/s-0038-1649503 29791947

[2] Flowers HL Skoretz SA Silver FL Rochon E Fang J Flamand-Roze C. Poststroke aphasia frequency, recovery, and outcomes: a systematic review and Meta-analysis. Arch Phys Med Rehabil. (2016) 97:2188–2201.e8. doi: 10.1016/j.apmr.2016.03.006, PMID: 27063364

[3] Baker C Worrall L Rose M Hudson K Ryan B O'Byrne L. A systematic review of rehabilitation interventions to prevent and treat depression in post-stroke aphasia. Disabil Rehabil. (2018) 40:1870–92. doi: 10.1080/09638288.2017.1315181, PMID: 28420284

[4] Boden-Albala B Litwak E Elkind MS Rundek T Sacco RL. Social isolation and outcomes post stroke. Neurology. (2005) 64:1888–92. doi: 10.1212/01.wnl.0000163510.79351.af 15955939

[5] Dalemans RJ de Witte L Wade D van den Heuvel W. Social participation through the eyes of people with aphasia. Int J Lang Commun Disord. (2010) 45:537–50. doi: 10.3109/13682820903223633, PMID: 19839875

[6] Naess H Hammersvik L Skeie GO. Aphasia among young patients with ischemic stroke on long-term follow-up. J Stroke Cerebrovasc Dis. (2009) 18:247–50. doi: 10.1016/j.jstrokecerebrovasdis.2008.10.005, PMID: 19560676

[7] Fridriksson J Hillis AE. Current approaches to the treatment of post-stroke aphasia. J Stroke. (2021) 23:183–201. doi: 10.5853/jos.2020.05015, PMID: 34102754 PMC8189855

[8] Brady MC Kelly H Godwin J Enderby P Campbell P. Speech and language therapy for aphasia following stroke. Cochrane Database Syst Rev. (2016) 2016:Cd000425. doi: 10.1002/14651858.CD000425.pub4, PMID: 27245310 PMC8078645

[9] Breitenstein C Grewe T Flöel A Ziegler W Springer L Martus P. Intensive speech and language therapy in patients with chronic aphasia after stroke: a randomised, open-label, blinded-endpoint, controlled trial in a health-care setting. Lancet. (2017) 389:1528–38. doi: 10.1016/s0140-6736(17)30067-3 28256356

[10] Kelly H Brady MC Enderby P. Speech and language therapy for aphasia following stroke. Cochrane Database Syst Rev. (2010) 5:Cd000425. doi: 10.1002/14651858.CD000425.pub2 20464716

[11] Berthier ML. Ten key reasons for continuing research on pharmacotherapy for post-stroke aphasia. Aphasiology. (2021) 35:824–58. doi: 10.1080/02687038.2020.1769987

[12] Norise C Hamilton RH. Non-invasive brain stimulation in the treatment of post-stroke and neurodegenerative aphasia: parallels, differences, and lessons learned. Front Hum Neurosci. (2016) 10:675. doi: 10.3389/fnhum.2016.00675, PMID: 28167904 PMC5253356

[13] Barker AT Jalinous R Freeston IL. Non-invasive magnetic stimulation of human motor cortex. Lancet. (1985) 325:1106–7. doi: 10.1016/s0140-6736(85)92413-4 2860322

[14] Rao J Li F Zhong L Wang J Peng Y Liu H. Bilateral cerebellar intermittent Theta burst stimulation combined with swallowing speech therapy for dysphagia after stroke: a randomized, double-blind, sham-controlled, Clinical Trial. Neurorehabil Neural Repair. (2022) 36:437–48. doi: 10.1177/15459683221092995, PMID: 35574927

[15] Szaflarski JP Nenert R Allendorfer JB Martin AN Amara AW Griffis JC. Intermittent Theta burst stimulation (iTBS) for treatment of chronic post-stroke aphasia: results of a pilot randomized, double-blind, Sham-Controlled Trial. Med Sci Monit. (2021) 27:e931468. doi: 10.12659/msm.931468, PMID: 34183640 PMC8254416

[16] Zhang JJ Bai Z Fong KNK. Priming intermittent Theta burst stimulation for Hemiparetic upper limb after stroke: a randomized controlled trial. Stroke. (2022) 53:2171–81. doi: 10.1161/strokeaha.121.037870, PMID: 35317611

[17] Chung SW Hill AT Rogasch NC Hoy KE Fitzgerald PB. Use of theta-burst stimulation in changing excitability of motor cortex: a systematic review and meta-analysis. Neurosci Biobehav Rev. (2016) 63:43–64. doi: 10.1016/j.neubiorev.2016.01.008, PMID: 26850210

[18] Allendorfer JB Nenert R Vannest J Szaflarski JP. A pilot randomized controlled trial of intermittent Theta burst stimulation as stand-alone treatment for post-stroke aphasia: effects on language and verbal functional magnetic resonance imaging (fMRI). Med Sci Monit. (2021b) 28:e934818. doi: 10.12659/msm.934818, PMID: 34862359 PMC8653428

[19] Chou TY Wang JC Lin MY Tsai PY. Low-frequency vs. Theta burst transcranial magnetic stimulation for the treatment of chronic non-fluent aphasia in stroke: a proof-of-concept study. Front Aging Neurosci. (2021) 13:800377. doi: 10.3389/fnagi.2021.800377, PMID: 35095477 PMC8795082

[20] Allendorfer JB Nenert R Nair S Vannest J Szaflarski JP. Functional magnetic resonance imaging of language following constraint-induced aphasia therapy primed with intermittent Theta burst stimulation in 13 patients with post-stroke aphasia. Med Sci Monit. (2021a) 27:e930100. doi: 10.12659/msm.930100, PMID: 33970893 PMC8120906

[21] Nettekoven C Volz LJ Kutscha M Pool EM Rehme AK Eickhoff SB. Dose-dependent effects of theta burst rTMS on cortical excitability and resting-state connectivity of the human motor system. J Neurosci. (2014) 34:6849–59. doi: 10.1523/jneurosci.4993-13.2014, PMID: 24828639 PMC4019799

[22] Li CT Chen MH Juan CH Huang HH Chen LF Hsieh JC. Efficacy of prefrontal theta-burst stimulation in refractory depression: a randomized sham-controlled study. Brain. (2014) 137:2088–98. doi: 10.1093/brain/awu109, PMID: 24817188

[23] Bozzay ML Brigido S van 't Wout-Frank M Aiken E Swift R Philip NS. Intermittent Theta burst stimulation in veterans with mild alcohol use disorder. J Affect Disord. (2021) 293:314–9. doi: 10.1016/j.jad.2021.06.039, PMID: 34229284 PMC8349789

[24] Zheng K Xu X Ji Y Fang H Gao F Huang G. Continuous theta burst stimulation-induced suppression of the right fronto-thalamic-cerebellar circuit accompanies improvement in language performance in poststroke aphasia: a resting-state fMRI study. Front Aging Neurosci. (2022) 14:1079023. doi: 10.3389/fnagi.2022.1079023 36711202 PMC9877515

[25] Geranmayeh F Chau TW Wise RJS Leech R Hampshire A. Domain-general subregions of the medial prefrontal cortex contribute to recovery of language after stroke. Brain. (2017) 140:1947–58. doi: 10.1093/brain/awx134, PMID: 29177494 PMC5903407

[26] Stockert A Wawrzyniak M Klingbeil J Wrede K Kümmerer D Hartwigsen G. Dynamics of language reorganization after left temporo-parietal and frontal stroke. Brain. (2020) 143:844–61. doi: 10.1093/brain/awaa023, PMID: 32068789

[27] Hertrich I Dietrich S Ackermann H. The role of the supplementary motor area for speech and language processing. Neurosci Biobehav Rev. (2016) 68:602–10. doi: 10.1016/j.neubiorev.2016.06.030 27343998

[28] Cheng L Xi H Gu H Gao Y Hu S Li M. Abnormalities of regional spontaneous brain activity in poststroke aphasia: a meta-analysis. Cereb Cortex. (2023) 33:7771–82. doi: 10.1093/cercor/bhad078, PMID: 36935094

[29] Xie X Hu P Tian Y Wang K Bai T. Transcranial alternating current stimulation enhances speech comprehension in chronic post-stroke aphasia patients: a single-blind sham-controlled study. Brain Stimul. (2022) 15:1538–40. doi: 10.1016/j.brs.2022.12.001, PMID: 36494053

[30] Carlino E Sigaudo M Pollo A Benedetti F Mongini T Castagna F. Nonlinear analysis of electroencephalogram at rest and during cognitive tasks in patients with schizophrenia. J Psychiatry Neurosci. (2012) 37:259–66. doi: 10.1503/jpn.110030, PMID: 22353633 PMC3380097

[31] Barbancho MA Berthier ML Navas-Sánchez P Dávila G Green-Heredia C García-Alberca JM. Bilateral brain reorganization with memantine and constraint-induced aphasia therapy in chronic post-stroke aphasia: an ERP study. Brain Lang. (2015) 145-146:1–10. doi: 10.1016/j.bandl.2015.04.003, PMID: 25932618

[32] Robson H Pilkington E Evans L DeLuca V Keidel JL. Phonological and semantic processing during comprehension in Wernicke's aphasia: an N400 and phonological mapping negativity study. Neuropsychologia. (2017) 100:144–54. doi: 10.1016/j.neuropsychologia.2017.04.012, PMID: 28433347

[33] Wilson KR O'Rourke H Wozniak LA Kostopoulos E Marchand Y Newman AJ. Changes in N400 topography following intensive speech language therapy for individuals with aphasia. Brain Lang. (2012) 123:94–103. doi: 10.1016/j.bandl.2012.06.005, PMID: 22944529

[34] Lucchese G Pulvermüller F Stahl B Dreyer FR Mohr B. Therapy-induced neuroplasticity of language in chronic post stroke aphasia: a mismatch negativity study of (a)grammatical and meaningful/less Mini-constructions. Front Hum Neurosci. (2016) 10:669. doi: 10.3389/fnhum.2016.00669, PMID: 28111545 PMC5216683

[35] Fritsch B Reis J Martinowich K Schambra HM Ji Y Cohen LG. Direct current stimulation promotes BDNF-dependent synaptic plasticity: potential implications for motor learning. Neuron. (2010) 66:198–204. doi: 10.1016/j.neuron.2010.03.035, PMID: 20434997 PMC2864780

[36] Wang HY Crupi D Liu J Stucky A Cruciata G Di Rocco A. Repetitive transcranial magnetic stimulation enhances BDNF-TrkB signaling in both brain and lymphocyte. J Neurosci. (2011) 31:11044–54. doi: 10.1523/jneurosci.2125-11.2011, PMID: 21795553 PMC3161730

[37] Yukimasa T Yoshimura R Tamagawa A Uozumi T Shinkai K Ueda N. High-frequency repetitive transcranial magnetic stimulation improves refractory depression by influencing catecholamine and brain-derived neurotrophic factors. Pharmacopsychiatry. (2006) 39:52–9. doi: 10.1055/s-2006-931542, PMID: 16555165

[38] Schulz KF Altman DG Moher D. CONSORT 2010 statement: updated guidelines for reporting parallel group randomised trials. J Pharmacol Pharmacother. (2010) 1:100–7. doi: 10.4103/0976-500x.72352, PMID: 21350618 PMC3043330

[39] Agha RA Altman DG Rosin D. The SPIRIT 2013 statement--defining standard protocol items for trials. Int J Surg. (2015) 13:288–91. doi: 10.1016/j.ijsu.2014.12.007, PMID: 25498499

[40] Mantovani A Rossi S Bassi BD Simpson HB Fallon BA Lisanby SH. Modulation of motor cortex excitability in obsessive-compulsive disorder: an exploratory study on the relations of neurophysiology measures with clinical outcome. Psychiatry Res. (2013) 210:1026–32. doi: 10.1016/j.psychres.2013.08.054, PMID: 24064461 PMC7325264

[41] Rossini PM Barker AT Berardelli A Caramia MD Caruso G Cracco RQ. Non-invasive electrical and magnetic stimulation of the brain, spinal cord and roots: basic principles and procedures for routine clinical application. Report of an IFCN committee. Electroencephalogr Clin Neurophysiol. (1994) 91:79–92. doi: 10.1016/0013-4694(94)90029-9, PMID: 7519144

[42] Kertesz A. Western aphasia battery--revised. San Antonio. TX: The Psychological Corporation. (2007). doi: 10.1037/t15168-000

[43] Hilari K Byng S Lamping DL Smith SC. Stroke and aphasia quality of life Scale-39 (SAQOL-39): evaluation of acceptability, reliability, and validity. Stroke. (2003) 34:1944–50. doi: 10.1161/01.str.0000081987.46660.ed, PMID: 12855827

[44] Hilari K Lamping DL Smith SC Northcott S Lamb A Marshall J. Psychometric properties of the stroke and aphasia quality of life scale (SAQOL-39) in a generic stroke population. Clin Rehabil. (2009) 23:544–57. doi: 10.1177/0269215508101729, PMID: 19447841

[45] Oldfield RC. The assessment and analysis of handedness: the Edinburgh inventory. Neuropsychologia. (1971) 9:97–113. doi: 10.1016/0028-3932(71)90067-4 5146491

[46] Goldstein LB Samsa GP. Reliability of the National Institutes of Health stroke scale. Extension to non-neurologists in the context of a clinical trial. Stroke. (1997) 28:307–10. doi: 10.1161/01.str.28.2.307 9040680

[47] Hong YH Wu SW Pedapati EV Horn PS Huddleston DA Laue CS. Safety and tolerability of theta burst stimulation vs. single and paired pulse transcranial magnetic stimulation: a comparative study of 165 pediatric subjects. Front Hum Neurosci. (2015) 9:29. doi: 10.3389/fnhum.2015.00029, PMID: 25698958 PMC4316715

[48] Duñabeitia JA Baciero A Antoniou K Antoniou M Ataman E Baus C. The multilingual picture database. Sci Data. (2022) 9:431. doi: 10.1038/s41597-022-01552-7, PMID: 35864133 PMC9304413

[49] Petrusic I Jovanovic V Kovic V Savic A. Characteristics of N400 component elicited in patients who have migraine with aura. J Headache Pain. (2021) 22:157. doi: 10.1186/s10194-021-01375-8, PMID: 34961473 PMC8903587

[50] Jia QX Su YY Liu G Chen ZY. Neurophysiological predictors of aphasia recovery in patients with large left-hemispheric infarction: a mismatch negativity study. Chin Med J. (2019) 132:2300–7. doi: 10.1097/cm9.0000000000000459, PMID: 31567479 PMC6819029

[51] Delorme A Makeig S. EEGLAB: an open source toolbox for analysis of single-trial EEG dynamics including independent component analysis. J Neurosci Methods. (2004) 134:9–21. doi: 10.1016/j.jneumeth.2003.10.009, PMID: 15102499

[52] Nicolo P Rizk S Magnin C Pietro MD Schnider A Guggisberg AG. Coherent neural oscillations predict future motor and language improvement after stroke. Brain. (2015b) 138:3048–60. doi: 10.1093/brain/awv200, PMID: 26163304

[53] Nicolo P Ptak R Guggisberg AG. Variability of behavioural responses to transcranial magnetic stimulation: origins and predictors. Neuropsychologia. (2015a) 74:137–44. doi: 10.1016/j.neuropsychologia.2015.01.033, PMID: 25619851

[54] Suppa A Huang YZ Funke K Ridding MC Cheeran B Di Lazzaro V. Ten years of Theta burst stimulation in humans: established knowledge, Unknowns and Prospects. Brain Stimul. (2016) 9:323–35. doi: 10.1016/j.brs.2016.01.006, PMID: 26947241

[55] Tsai YC Li CT Liang WK Muggleton NG Tsai CC Huang NE. Critical role of rhythms in prefrontal transcranial magnetic stimulation for depression: a randomized sham-controlled study. Hum Brain Mapp. (2022) 43:1535–47. doi: 10.1002/hbm.25740, PMID: 34873781 PMC8886663

[56] Singh A Erwin-Grabner T Sutcliffe G Paulus W Dechent P Antal A. Default mode network alterations after intermittent theta burst stimulation in healthy subjects. Transl Psychiatry. (2020) 10:75. doi: 10.1038/s41398-020-0754-5, PMID: 32094326 PMC7040002

[57] Nachev P Kennard C Husain M. Functional role of the supplementary and pre-supplementary motor areas. Nat Rev Neurosci. (2008) 9:856–69. doi: 10.1038/nrn2478 18843271

[58] Dick AS Bernal B Tremblay P. The language connectome: new pathways, new concepts. Neuroscientist. (2014) 20:453–67. doi: 10.1177/1073858413513502 24342910

[59] Thiebaut de Schotten M Dell'Acqua F Valabregue R Catani M. Monkey to human comparative anatomy of the frontal lobe association tracts. Cortex. (2012) 48:82–96. doi: 10.1016/j.cortex.2011.10.001, PMID: 22088488

[60] Ziegler W Kilian B Deger K. The role of the left mesial frontal cortex in fluent speech: evidence from a case of left supplementary motor area hemorrhage. Neuropsychologia. (1997) 35:1197–208. doi: 10.1016/s0028-3932(97)00040-7, PMID: 9364490

[61] Fisicaro F Lanza G Grasso AA Pennisi G Bella R Paulus W. Repetitive transcranial magnetic stimulation in stroke rehabilitation: review of the current evidence and pitfalls. Ther Adv Neurol Disord. (2019) 12:1756286419878317. doi: 10.1177/1756286419878317, PMID: 31598137 PMC6763938

[62] Dalton SGH Cavanagh JF Richardson JD. Spectral resting-state EEG (rsEEG) in chronic aphasia is reliable, sensitive, and correlates with functional behavior. Front Hum Neurosci. (2021) 15:624660. doi: 10.3389/fnhum.2021.624660, PMID: 33815079 PMC8010195

[63] Chang C-T Lee C-Y Chou C-J Fuh J-L Wu H-C. Predictability effect on N400 reflects the severity of reading comprehension deficits in aphasia. Neuropsychologia. (2016) 81:117–28. doi: 10.1016/j.neuropsychologia.2015.12.002, PMID: 26686551

[64] Balkowiec A Katz DM. Cellular mechanisms regulating activity-dependent release of native brain-derived neurotrophic factor from hippocampal neurons. J Neurosci. (2002) 22:10399–407. doi: 10.1523/jneurosci.22-23-10399.2002, PMID: 12451139 PMC6758764

[65] Lee CW Chu MC Wu HF Chung YJ Hsieh TH Chang CY. Different synaptic mechanisms of intermittent and continuous theta-burst stimulations in a severe foot-shock induced and treatment-resistant depression in a rat model. Exp Neurol. (2023) 362:114338. doi: 10.1016/j.expneurol.2023.114338, PMID: 36717014

[66] Wang L Zhou Y Chen X Liu J Qin X. Long-term iTBS promotes neural structural and functional recovery by enhancing neurogenesis and migration via miR-551b-5p/BDNF/TrkB pathway in a rat model of cerebral ischemia-reperfusion injury. Brain Res Bull. (2022) 184:46–55. doi: 10.1016/j.brainresbull.2022.03.002, PMID: 35257808

[67] Jannati A Oberman LM Rotenberg A Pascual-Leone A. Assessing the mechanisms of brain plasticity by transcranial magnetic stimulation. Neuropsychopharmacology. (2023) 48:191–208. doi: 10.1038/s41386-022-01453-8, PMID: 36198876 PMC9700722

[68] Velioglu HA Hanoglu L Bayraktaroglu Z Toprak G Guler EM Bektay MY. Left lateral parietal rTMS improves cognition and modulates resting brain connectivity in patients with Alzheimer's disease: possible role of BDNF and oxidative stress. Neurobiol Learn Mem. (2021) 180:107410. doi: 10.1016/j.nlm.2021.107410, PMID: 33610772

[69] Bai G Jiang L Ma W Meng P Li J Wang Y. Effect of low-frequency rTMS and intensive speech therapy treatment on patients with nonfluent aphasia after stroke. Neurologist. (2020) 26:6–9. doi: 10.1097/nrl.0000000000000303, PMID: 33394904

